# Biosensing by Polymer-Coated Etched Long-Period Fiber Gratings Working near Mode Transition and Turn-around Point

**DOI:** 10.3390/bios13070731

**Published:** 2023-07-13

**Authors:** Tanoy Kumar Dey, Cosimo Trono, Palas Biswas, Ambra Giannetti, Nandini Basumallick, Francesco Baldini, Somnath Bandyopadhyay, Sara Tombelli

**Affiliations:** 1 Central Glass and Ceramic Research Institute, CSIR-CGCRI, 196 Raja S C Mullick Road, Kolkata 700032, India; tanoykumardey@gmail.com (T.K.D.); palas@cgcri.res.in (P.B.); nandini_b@cgcri.res.in (N.B.); 2 Istituto di Fisica Applicata “Nello Carrara”, CNR-IFAC, Via Madonna del Piano 10, 50019 Sesto Fiorentino, Italy; a.giannetti@ifac.cnr.it (A.G.); f.baldini@ifac.cnr.it (F.B.); s.tombelli@ifac.cnr.it (S.T.)

**Keywords:** long-period fiber grating, label-free biosensing, etching, cladding mode, sensitivity enhancement

## Abstract

A methodology to enhance the sensitivity of long-period fiber gratings (LPFGs) based on the combination of three different enhancement approaches is presented; the methods here adopted are the working near mode transition (MT) of a cladding mode (CM), working near the turn-around point of a CM and the enhancement of the evanescent field of CMs by reducing the cladding diameter or by increasing the order number of CMs. In order to combine these enhancement methodologies, an electrostatic self-assembly (ESA) process was used to deposit a polymeric overlay, with a chosen thickness, onto the etched fiber. The add-layer sensitivity of the sensor was theoretically calculated, and the demonstration of the real applicability of the developed LPFG as a biosensor was performed by means of an IgG/anti-IgG immunoassay in human serum in a thermostated microfluidic system. The limits of detection (LODs) calculated by following different procedures (three times the standard deviation of the blank and the mean value of the residuals) were 6.9 × 10^−8^ µg/mL and 4.5 × 10^−6^ µg/mL, respectively. The calculated LODs demonstrate the effectiveness of the applied methodology for sensitivity enhancement.

## 1. Introduction

Long-period fiber gratings (LPFGs) have been widely used to develop chemo- and biosensors throughout the last decade, thanks to their ability to sense surrounding refractive index (SRI) variations [1,2,3,4]. Despite their success in the field, meeting the sensitivity requirements to detect biomolecules in real samples is still a challenge to be addressed due to the LPFG lower sensitivity around SRI 1.333 [3], which is the proper RI of solutions and samples commonly involved in biosensing applications (e.g., RI of buffers) [5,6,7]. To this purpose, different methodologies have been applied by researchers to enhance the SRI sensitivity of the sensor in this range; these methods can be broadly classified into three main categories:

(a) Working near mode transition (MT) of a cladding mode (CM) [5,8,9,10].
(b) Working near the turn-around point (TAP) of a CM [11,12].
(c) Enhancement of the evanescent field of CMs by reducing the cladding diameter or by increasing the order number of CMs [11,13,14].

Sometimes, a combination of these methodologies has been used to enhance the SRI sensitivity [15,16,17,18,19]. It has been theoretically shown that by combining all these methodologies, an SRI sensitivity of 1.43 × 10^5^ nm/SRIU (SRI units) is achievable [20].

However, it is worth mentioning here that characterizing an LPFG sensor in terms of its sensitivity to bulk SRI variations is not fully appropriate for biosensing applications in which the sensing mechanism is usually based on the binding of the analytes of interest directly onto the functionalized surface of the fiber. It is apparent that an increase in the penetration depth of the evanescent wave of the cladding modes in the surrounding environment does not lead automatically to an improvement of the biosensing performances. The key parameter is the ratio between the penetration depth of the evanescent wave and the thickness of the region where the chemical/biochemical interaction takes place. The larger the value of this ratio, the smaller the fraction of the evanescent wave, which is modulated by the analyte to be measured, and this leads to a worsening of the sensor performance, notwithstanding an increase in the bulk SRI sensitivity. For this reason, it is usually more significant to characterize LPFG sensors in terms of add-layer sensitivity. In Bandyopadhyay et al. [21], a theoretical study on the optimization of LPFG sensor add-layer sensitivity is reported, while the design and experimental validation of an LPFG sensor working near TAP of a CM and working near MT of a CM is described in Dey et al., 2021 [22] and 2022 [23], respectively.

In this work, for the first time and to the best of our knowledge, we theoretically designed and practically realized an LPFG biosensor by combining all three sensitivity enhancement methodologies. The period of the grating is optimized in such a way that the left peak of the resonant CM of our interest could be positioned in the C+L band (1530–1625 nm) by combining, at the same time, the enhancement conditions of the evanescent field, working near TAP and working near MT. To work near MT, a layer-by-layer method employing an electrostatic self-assembly (ESA) process is used to deposit a polymeric overlay (RI~1.53) constituted by multiple layers of poly(allylamine hydrochloride) and poly(sodium 4-styrenesulfonate) onto the sensor surface. The add-layer sensitivity of the sensor was theoretically calculated; then, an IgG/anti-IgG immunoassay was performed in serum inside a thermally stabilized closed-flow cell. A calibration curve of the immunosensor was achieved in order to evaluate the real effectiveness and feasibility of the sensor, and this result was compared with other LPFG-based sensors with application in IgG/anti-IgG detection to prove the effectiveness of the sensitivity enhancement technique.

## 2. Materials and Methods

### 2.1. Materials

Reagents for sensor modification and assay implementation—hydrofluoric acid (HF), goat anti-mouse IgG, bovine serum albumin (BSA), reagent for polymer coating poly(allylamine hydrochloride) (PAH) and poly(sodium 4-styrenesulfonate) (PSS)—and the reagents for phosphate buffer saline (PBS, 0.01 M pH 7.4) preparation were all from Merck Life Science (Milan, Italy). Mouse IgG, 1 ethyl—3(3- dimethyl amino propyl) carbodiimide HCl (EDC) and N- hydroxy succinimide (NHS) were purchased from Thermo Fisher Scientific (Milan, Italy). Pooled normal human serum was from HyTest Ltd. (Turku, Finland).

### 2.2. Inscription of LPFG Sensor

The LPFG was written on B/Ge co-doped photosensitive fiber Fibercore PS 1250/1500 (cutoff wavelength at 1209 nm) using a KrF excimer laser (Compex 110, Lambda Physics GmbH, Gottingen, Germany). The acrylate coating of the fiber was removed along a 40 mm section before the inscription. A micrometric slit was used for the shaping of the laser beam in order to obtain a 50% duty cycle of the induced RI change profile. A cylindrical lens of focal length 100 mm was used for increasing the laser fluence (the fiber was placed 1 cm away from the lens focus). The total pulses per grating plane were 250 and the total fluence per grating plane was 70 J/cm^2^. The LPFG was placed in the central part of the stripped region of the fiber.

### 2.3. Etching Process

A chemical etching process was used for the reduction of the cladding diameter by using 10% and 1% HF. The fiber was kept straight during the etching process to remove the bend-induced noise by fixing it in a U-shaped holder by using small magnetic blocks. During the whole process, the fiber was never touched. The details of the etching setup can be found in Dey et al., 2021 [24]. After etching, the fiber was examined under a microscope (Inverted fluorescence microscope, Zeiss Axio Observer.Z1, Zeiss, Jena, Germany) to measure the actual diameter.

### 2.4. Polymer-Coating Process

The electrostatic self-assembly (ESA) technique was used to deposit the polymer overlay on the fiber surface. The process was described in detail in Dey et al. 2022 [22]. During the coating, the fiber was kept straight by using the same U-shaped holder of the etching setup. The fiber surface was cleaned using deionized water followed by methanol. Then, the surface was treated with a 1 M sodium hydroxide (NaOH) solution to form a silanol group and was rinsed with a copious amount of water. After surface preparation, the fiber was alternatively dipped in poly-cation and poly-anion solutions to obtain the desired amount of polymer thickness. In this experiment, PAH and PSS were used as poly-electrolytes. One combined layer of PAH/PSS was denoted as a bilayer, and the thickness of each bilayer was ~28 nm [23]. The thickness of the polymeric bilayer depends on the salt concentration of the polyelectrolyte solution [24], the rinsing procedure after polyelectrolyte deposition [25], the pH of the polyelectrolyte solution [26], etc. It also depends on the considered polyelectrolytes. In [26], it was shown that a bilayer thickness of ~28 nm can be obtained with PAH/PSS with the controlled pH of a PSS solution. The outer surface of the polymeric layer was of PAH as it contains amine (-NH_2_) groups, which are needed for the covalent binding of the biomolecule on the fiber surface. The RI of the polymeric layer is ~1.53 [23].

### 2.5. Immunoassay Protocol

**Figure 1:**
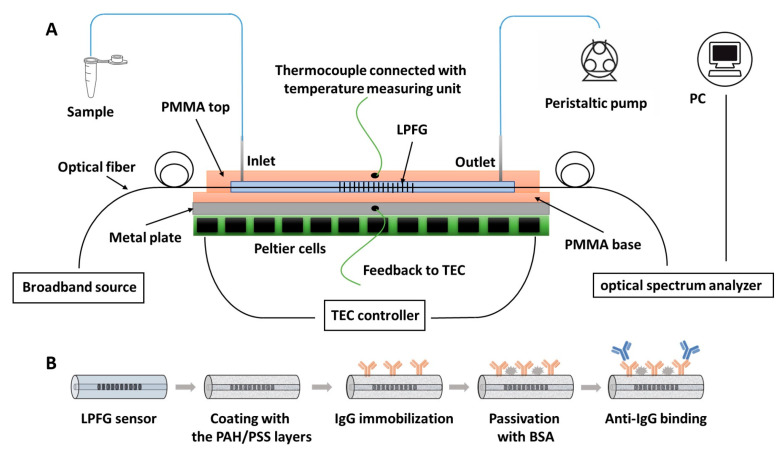
(**A**) Scheme of the experimental setup. (**B**) Scheme of the immunoassay conducted on the long-period fiber grating (LPFG) sensor.

For the immunoassay, the coated fiber was fixed into a thermally stabilized closed flow cell [27] at a constant temperature of 25 ± 0.1 °C by means of a set of three Peltier cells connected in series and a thermocouple for the temperature monitoring (LDC-3722B thermoelectric cooler (TEC) controller, ILX Lightwave, MT, USA). A peristaltic pump was used to flow the different solutions needed for the preparation of the biomolecular layer and for the antigen interaction. A scheme of the experimental setup is shown in Figure 1A together with the scheme of the immunoassay conducted on the LPFG sensor (Figure 1B).

The coated fiber fixed into the flow cell was then modified with mouse IgG, which is used as a biorecognition element to bind the specific target antibody (anti-mouse IgG). For the immobilization, the carboxylic groups of the antibody (mouse IgG) were first activated by classical crosslinking chemistry (EDC and NHS); then, the activated antibody (1000 µg/mL in PBS) was flowed into the cell at a flow rate of 0.9 μL/min for 1 h. After washing with PBS to remove the unbound biomolecules from the surface, the surface was passivated with BSA (3% in PBS, at a flow rate of 6.75 μL/min for 30 min) to prevent nonspecific adsorption.

The assay was performed with increasing concentrations of goat anti-mouse IgG ranging from 0.1 ng/mL to 100 µg/mL, spiked in human serum (at a final dilution of 1:10 (*v*/*v*) in PBS). Human serum was used as a complex matrix to demonstrate the possibility of using the developed biosensors in real applications and as a negative control to demonstrate the biosensor specificity.

The injection procedure was the following:

1. Fast injection at 45 μL/min for 3 min (fast filling of the flow cell).
2. Slow flow at 6.75 μL/min for ~20 min (binding phase at slow flow rate).
3. PBS washing after each step at a flow rate of 45 μL/min for 5 min.

The acquisition of the resonant wavelength of a CM of LPFG (*λ_res_*) for every anti-IgG concentration was performed in stop-flow condition and in PBS medium for 5 min.

### 2.6. Data Acquisition and Analysis

The optical source and detection system were the broadband SLED SLD-1310/1430/1550/1690-10 (FiberLabs Inc., Saitama, Japan) and the optical spectrum analyzer (OSA) MS9030A/9701C (Anritsu, Kanagawa, Japan), respectively. The measurement spectral range ranged from 1300 nm up to 1750 nm. The interaction of the optical signal with the grating is characterized by the presence of dips on the transmitted spectrum. The dips were fitted with a Lorentzian function for the calculation of the minimum wavelength (the resonance wavelength, indicated as *λ_res_* in Section 3.1). Every experimental point was calculated as the average of 25 samples (*λ_res_*) acquired in stop-flow conditions.

## 3. Results and Discussion

### 3.1. Theory and Simulation

LPFG couples the fundamental core mode (LP_0,1_ mode) to copropagate higher-order CMs (LP_0,m_ modes where m = 2, 3, 4, …….) at different wavelengths (*λ_res_*), where the following resonant condition is satisfied:

(1) $$\lambda_{res}=\left(n_{eff}^{co}-n_{eff}^{cl0,m}\right)\Lambda$$

where $n_{eff}^{co}$ is the effective refractive index of the core mode, $n_{eff}^{cl0,m}$ is the effective refractive index of m^th^-order CM and Λ is the period of the grating [28].

It is known that LPFGs attain very high SRI sensitivity near the TAP of a CM [11], and, in our previous work [23], we already showed that by reducing the cladding diameter, a lower-order dispersed CM near TAP became more sensitive with respect to higher-order CMs in a non-etched fiber because of the enhancement of the evanescent field of the CM as a result of the reduction in the cladding diameter. Working near MT is another way to enhance the SRI sensitivity: it consists of the deposition of an overlay layer of any dielectric, metal or metal–dielectric composite having an RI higher than the core RI on the fiber surface up to a certain thickness, known as optimum overlay thickness (OOT) [8]. It is worth mentioning here that the MT of all CMs occurs for the same thickness variation of a certain overlay material [8]. During the bioassay implementation, the bulk SRI remains constant (buffer of RI ~1.333), while the local surface RI and the thickness of the biomolecular layer variation give rise to the resonant wavelength shift of the CMs. In order to determine the add-layer thickness range for which the sensitivity is linear and maximum (the central thickness value is the so-called OOT) [23], the sensor was here characterized in terms of add-layer sensitivity (nm_WL_/nm_TH_ = wavelength shift in nanometer per nanometer thickness variation on sensor surface [21]).

A theoretical simulation was carried out to optimize the design parameters of the LPFG sensor and subsequently to enhance its add-layer sensitivity by exploiting the combined effect of mode dispersion through fiber etching, operating the CM around the linear part of the MT and also around the TAP. The concept of dispersing a cladding mode to its TAP has already been described earlier, where the lowest order LP_0,2_ cladding mode was dispersed to its TAP. Although the add-layer sensitivity could be significantly increased, the diameter of the fiber after etching was found to be around 20 μm [27]. In real-world applications, using a fiber of that size is difficult. It should be mentioned at this point that any mode order can be dispersed through etching to their respective TAPs, but, considering that the higher mode order needs a lower cladding reduction but exhibits a lower sensitivity after etching, there is a need for a trade-off between the choice of a mode order to be dispersed through etching and the final cladding diameter. There is another important factor to consider when selecting a specific cladding mode, which is the final location of the dual resonant bands of the cladding mode once it is dispersed to its TAP. The purpose of operating a cladding mode around the TAP is to take advantage of the possibility of monitoring the shift of both bands in order to improve measurement resolution. To achieve that, the dual bands should be positioned within the available source bandwidth of commercial wideband optical sources, which mostly covers the C and extended L bands and, in general, covers a band from 1520 nm to 1750 nm. Taking the design constraints discussed above into account, the LP_0,7_ cladding mode was considered in our simulation to realize the sensor. The parameters used for the simulation are shown in Table 1.

**Figure 2:**
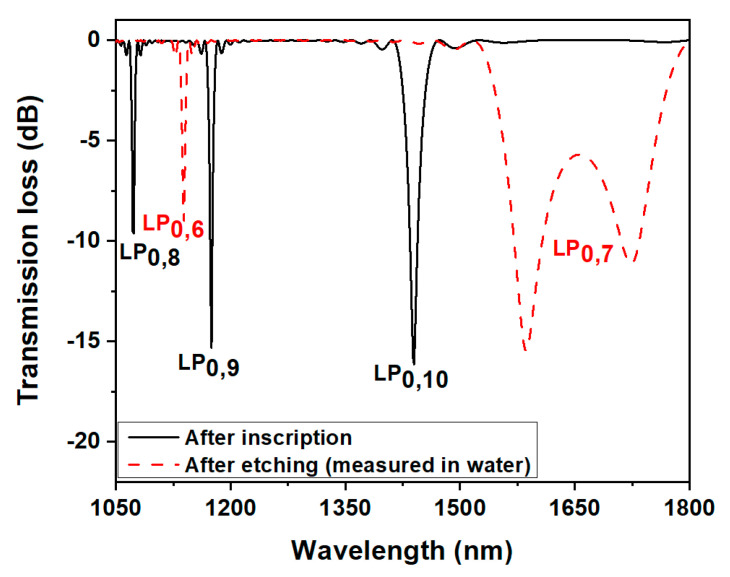
Simulated spectrum of the LPFG after inscription and after etching up to 84.9 µm.

The resonant spectrum of the grating was simulated over a wide band (1050 nm to 1800 nm), considering water as the surrounding medium (RI = 1.334). With these grating parameters, the left peak of the LP_0,8_, LP_0,9_ and LP_0,10_ cladding modes could be seen within the considered bandwidth (Figure 2). The fiber diameter was then reduced in steps, and spectra were computed for every step, up to a cladding diameter of 84.9 µm, where the LP_0,7_ cladding mode could be dispersed near its TAP within the desired wavelength band ranging from 1520 to 1750 nm (Figure 2). At this point, it can be said that the LP_0,7_ mode was positioned near its TAP in a water medium by dispersing the mode. It was then required to compute the spectrum while it was positioned near the linear part of its mode transition.

It is important to mention that while tailoring the spectral characteristics of a cladding mode, either by etching the fiber or by deposition of overlay layers, it is necessary to precisely position the phase matching curve (PMC) of that respective cladding mode [12,21]. It was observed earlier that positioning a cladding mode around the start of the linear region of its MT curve needs a nearly 250 nm thick overlay (with RI ≃ 1.53 RIU) layer on the surface of the grating [22]. It was also observed that upon deposition of a high refractive index overlay layer, the resonant cladding mode spectrum is redshifted if the PMC of that CM has a negative slope and similarly experiences a blueshift if the slope of the PMC is positive. Now, among the two resonant bands of a cladding mode positioned near the TAP, the resonant band that is located on the right-hand side of the TAP (namely, the right peak) moves on the section of the PMC, which has a negative slope, while the left peak moves on the positive PMC section. It is therefore understandable that by deposition of a ~250 nm overlay layer of a polymer (RI = 1.53), although the mode will be positioned near the MT, the dual resonant bands of LP_0,7_ mode as computed and shown in Figure 2 will move away from the TAP, and, consequently, the sensitivity will be significantly reduced. Therefore, the PMC of the LP_0,7_ mode has to be pre-processed so that even though the overlay layer of a desired material and thickness is deposited to position the cladding mode near the MT, the condition of operating the same mode near the TAP does not get affected. To achieve this optimal condition, the initial PMC was tailored by reducing the diameter of the fiber from 84.9 µm in a step of 0.01 µm, and at each step, the spectrum of the LP_0,7_ mode was recomputed after incorporating a layer of a thickness of 266 nm with RI = 1.53 RIU, which is necessary to position the CM at MT. With the reduction in cladding diameter, the dual resonant bands gradually come closer, then converge to a single band and, with a further reduction in cladding diameter, finally vanish [12].

**Figure 3:**
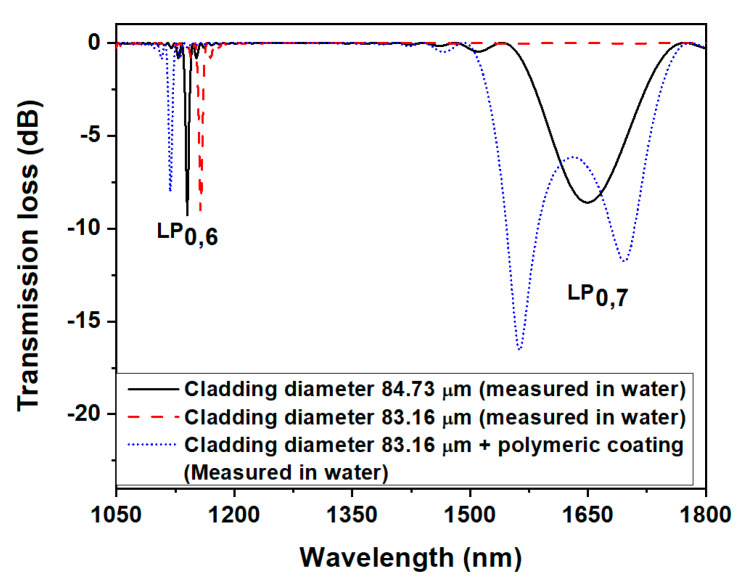
Simulated spectrum of the LPFG after etching and 266 nm layer (refractive index (RI) 1.53) deposition.

Figure 3 describes the results for the present simulation, where, amongst all computed spectra of LP_0,7_, only spectra at two different cladding diameters are plotted as an example. When the cladding diameter is reduced to 84.73 μm, the two peaks merge into a single one (black line in Figure 3), and at a diameter of 83.16 μm, the phase-matching condition for the LP_0,7_ mode is not satisfied, and no resonant band can be observed (red dashed line). The deposition of a 266 nm overlay layer (RI = 1.53) brings again the dual resonant bands to have a peak-to-peak separation of 120 nm (blue dotted line in Figure 3).

It is worth mentioning here that a further reduction in the cladding diameter beyond 83.16 µm implies a deposition of a thicker polymer layer on the sensor surface to regain the dual resonance of LP_0,7_ CM near TAP, with a decrease in the final sensitivity.

**Figure 4:**
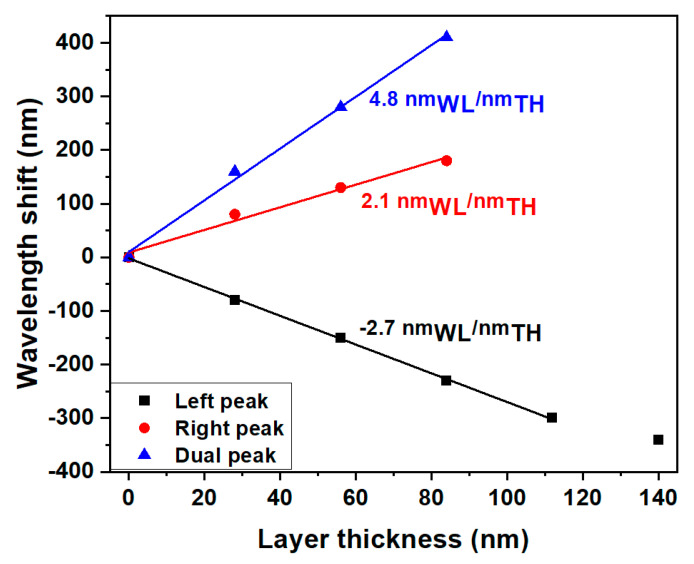
Calculated add-layer sensitivity of the sensor at the point of operation considering a layer with RI = 1.53.

The add-layer sensitivity of the sensor was calculated by increasing the layer thickness on the optimized sensor surface with a sequence of layers of a material with RI = 1.53 (the same RI used to bring the sensor to the MT region). Each wavelength shift was simulated by the deposition of 5 layers one by one (each 28 nm thick). The theoretical response curve is depicted in Figure 4. It was found that the wavelength shift of the left peak remains constant for the first four layers and then decreases. This was because, after deposition of 112 nm thickness (28 nm × 4 layers), the LP_0,7_ CM exceeds the linear part of the MT curve. The right peak after the deposition of three layers was not considered since it is beyond the wavelength range of the OSA later used in the experiments. The calculated sensitivity in the linear part was −2.7 nm_WL_/nm_TH_ for the left peak and 2.1 nm_WL_/nm_TH_ for the right peak, and the dual peak sensitivity became 4.8 nm_WL_/nm_TH_.

### 3.2. Fabrication of the LPFG Sensor

**Figure 5:**
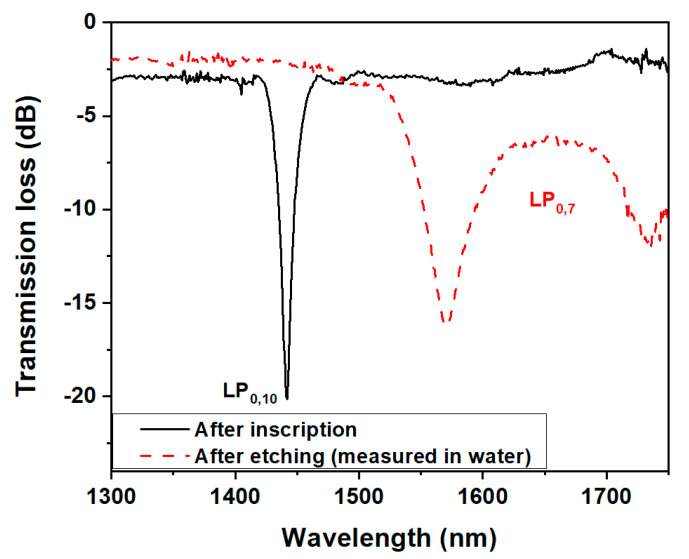
LPFG spectra after inscription (black line) and after etching with 10% HF (red dashed line).

The LPFG was fabricated by following the results derived from the simulation, as described in Section 2.2. After inscription, the cladding diameter was reduced by the chemical etching process described in Section 2.3, until the dual peak resonance of the LP_0,7_ CM appeared near the TAP in a water medium. The experimental transmission spectra before and after the etching process are depicted in Figure 5. The left peak of the mode appeared at ≃1570 nm with a peak-to-peak separation of ≃155 nm, which matches the simulation results described in the previous section well.

**Figure 6:**
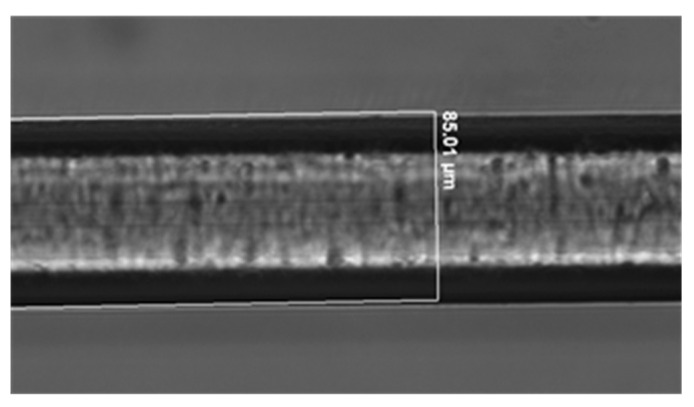
Microscopic image of the etched fiber.

The diameter of the fiber measured by using the microscope was 85.01 µm (Figure 6), which was in accordance with the simulation results.

**Figure 7:**
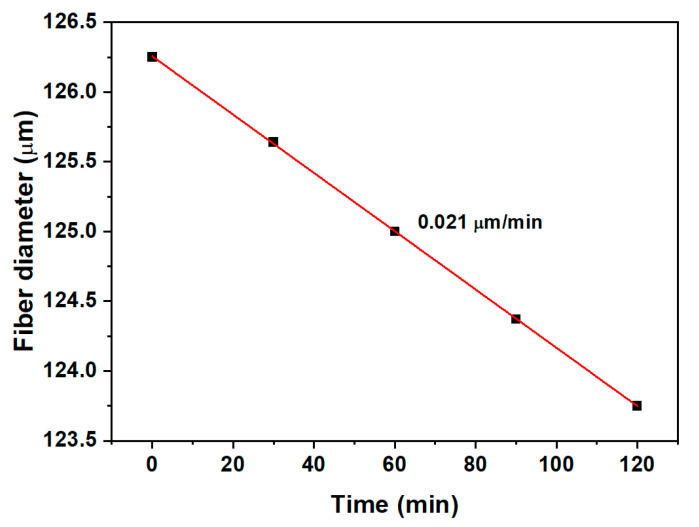
Etching rate calculation of Fibercore PS 1250/1500 fiber using 1% HF.

**Figure 8:**
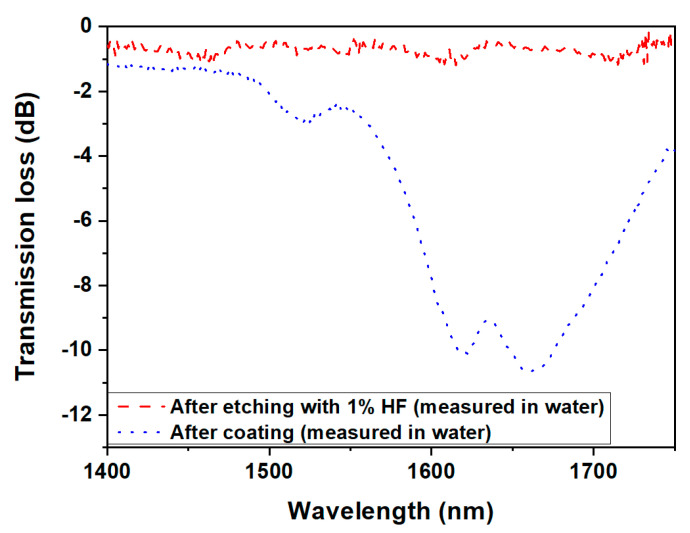
Spectra of LPFG after etching using 1% HF and after polymer coating.

As derived from the simulation, to introduce the MT condition along with the TAP condition and mode dispersion, the fiber diameter must be further reduced by 1.74 µm so that, after polymeric layer deposition of a thickness of 250 nm, the dual resonance of LP_0,7_ CM can appear again near the TAP. In order to find the correct protocol (etching time and HF dilution) for this subtle thickness reduction, a dummy Fibercore PS 1250/1500 fiber was etched in 1% HF solution. After every 30 min, the fiber was taken out of the HF solution, and the diameter was measured using the microscope. The total etching duration was 120 min, and the etching rate, calculated from corresponding data, was 0.021 μm/min, as shown in Figure 7. Considering these results, the LPFG sensor of diameter ~85.01 μm was immersed in the 1% HF solution for 83 min for a further reduction in the fiber diameter by ~1.74 μm. The corresponding spectrum is shown in red in Figure 8.

After this further etching step, the polymer layer was deposited on the LPFG surface by using the ESA method as described in Section 2.4. Nine bilayers and a final monolayer of PAH were deposited on the sensor for a total polymer thickness of ≃266 nm, with the thickness of a single PAH/PSS bilayer being equal to ≃28 nm (well matched with the value considered during simulation) and assuming the thickness of the PAH monolayer to be equal to 14 nm. As a result, the dual peak of the LP_0,7_ CM appeared again near the TAP in a water medium as shown in Figure 8 (blue line). The wavelength of the left peak of the dual resonance was ≃1619 nm with a peak-to-peak separation of ≃50 nm. The slight mismatch with simulation results, which led to a left peak at 1565 nm and a peak-to-peak separation of 130 nm, can be ascribed to a slight discrepancy between the experimental and calculated final diameter of the etched fiber. In any case, the LP_0,7_ CM of the sensor is near the TAP, along with the MT condition and with the enhanced evanescent field obtained due to mode dispersion in the water medium.

### 3.3. Immunoassay to Evaluate the Sensor Performance

With the final aim of demonstrating the real applicability of the developed LPFG as a biosensor, an immunoassay was implemented on its surface in order to investigate the analytical improvements of the new sensor amplification strategy. In order to be able to perform a correct comparison with previous works based on modified LPFG-based biosensors for the detection of immunoglobulins G [29,30,31,32,33,34,35,36,37,38], an IgG/anti-IgG immunoassay was accomplished, and the results were used to evaluate the sensitivity in terms of the limit of detection (LOD).

**Figure 9:**
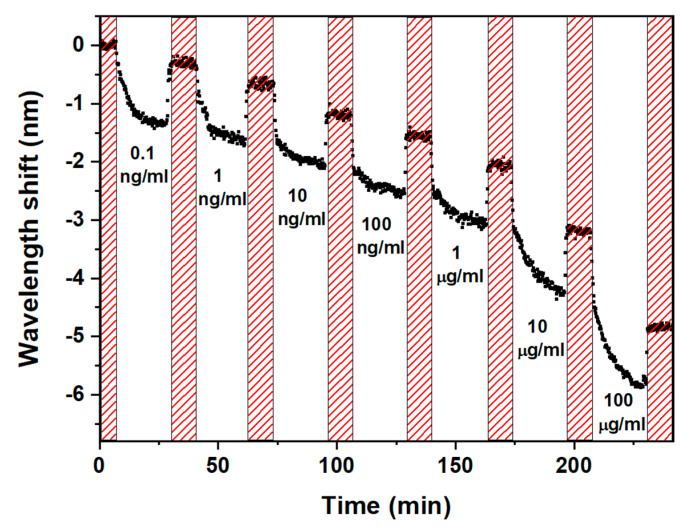
Sensorgram of the left peak of LP_0,7_ CM during the assay.

**Figure 10:**
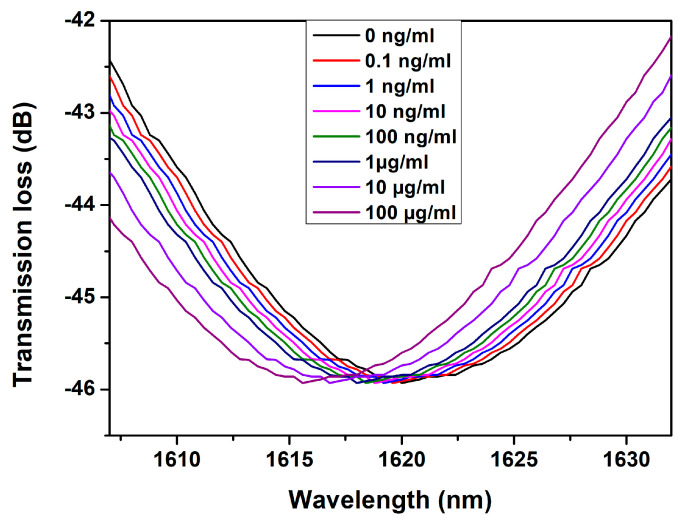
Spectra of the left peak of LP_0,7_ CM during the assay.

The immunoassay was performed as described in Section 2.5. The real-time sensorgram of the left peak of the LP_0,7_ CM is shown in Figure 9. The shaded region in Figure 8 represents the PBS washing and the stop-flow phase. The corresponding spectra of the left peak of the LP_0,7_ CM at the stop-flow phase are shown in Figure 10.

**Figure 11:**
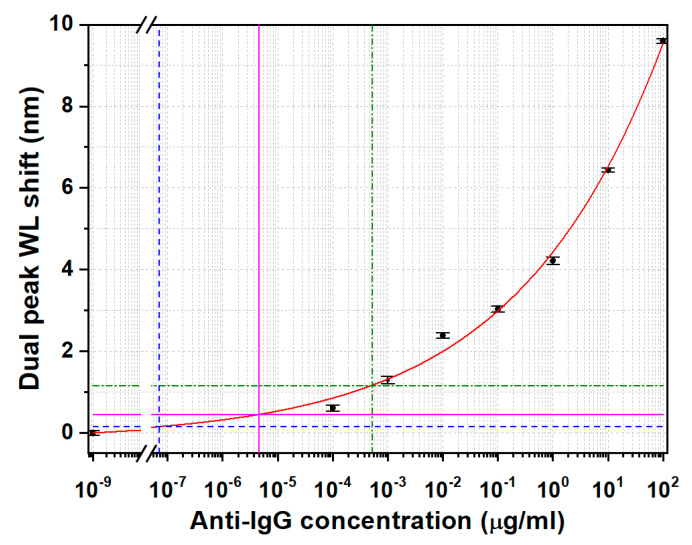
Dual peak resonant wavelength shifts of the LP_0,7_ CM at different concentrations of antigen. Dashed blue line: limit of detection (LOD) calculated by using three times the standard deviation of the blank. Green dashed-dotted line: LOD calculated by using the maximum of the residuals. Magenta line: LOD calculated by using mean value of the residuals.

The dual peak resonance wavelength shifts in correspondence with the different concentrations of goat anti-mouse IgG are reported in Figure 11. The error bars shown here are calculated as the standard deviation on 25 acquired wavelengths. The experimental data were fitted by means of a logistic sigmoidal model [39] described by the following model equation (Equation (2)):

(2) y=A1−A21+(xx0)p+A2

The parameters obtained by means of the fitting procedure and the data of Figure 11 are summarized in Table 2.

Despite the fact that the correlation between experimental data and the fit is very good (R-square = 0.996), the parameters A_2_ (the asymptote) and x_0_ (middle point between A_1_ and A_2_) of the logistic function are affected by big errors. This can be explained considering that the logistic curve identified by Equation (2) is a sigmoidal curve with a horizontal asymptote A_2_, which represents the saturation of the biochemical capture layer onto the fiber surface, and a flex, close to the x_0_ middle point. In this case, the fiber surface is far from being saturated, as demonstrated by the fact that the flex point has not yet been reached in the fit curve reported in Figure 9, with the consequence that a little variation in the experimental points can also induce a large variation in the fitting curve parameters (large errors on x_0_ and A_2_). However, this model function was chosen here because it has been reported to be well correlated with biochemical phenomena [39]. Moreover, it can be confidently used for the estimation of the LOD because of the optimal correlation with the experimental data: by following the formal definition of the LOD of three times the standard deviation of the blank [40], which, in this case, is the anti-IgG zero concentration, we obtain a LOD of 6.9 × 10^−8^ µg/mL (dashed blue lines in Figure 9). However, after a critical analysis of the data and, in particular, of the residuals (the distance of the data from the fitted curve, which is, in turn, the difference between the measured value and the predicted value), it is evident that two points deviate from the fitting curve. For this reason, different protocols were also followed to calculate the LOD by considering the maximum of the residuals (LOD = 5.1 × 10^−4^ µg/mL, green dashed-dotted line in Figure 11) and the mean value of the residuals (LOD = 4.5 × 10^−6^ µg/mL, magenta line in Figure 11) to take into consideration these deviations.

The proposed biosensor has the potential of reaching a LOD of 6.9 × 10^−8^ µg/mL, after understanding the source of noise that caused the data to deviate from the theoretical ideal trend. However, for a more unbiased comparison with previous works (Table 3) based on the same measurement principle and applied to the detection of the same biomolecules, the two LODs calculated from the residuals are considered. Moreover, these LOD values can be deemed as more reliable since they are within or near the range of tested concentrations.

As evidenced in Table 3, the reached LOD calculated with the stricter method (the maximum value of the residuals) is of the same order as the one found in Ref. [36] and still one of the best, despite the severity of the calculation procedure. On the other hand, with the less harsh method (based on the average of the residuals), an improvement of two orders of magnitude was obtained in the LOD, still at a reasonable distance from the lowest tested concentration. It should be noted that these excellent performances were reached in the detection of the target antibody spiked in serum, which is a very complex matrix and also proves the specificity of the developed LPFG biosensor. Further advantages are provided by the use of a temperature-controlled flow cell, which makes the biosensor more user-friendly and less prone to interferences from environmental external factors such as temperature and strain.

Summarizing, we can affirm that the combination of the three sensitivity enhancement techniques, together with proper control of the working conditions by the use of the temperature-controlled flow cell, can lead to a very good improvement in the detection limits in immunoassays by LPFGs even when not considering the more advantageous calculation method based on three times the standard deviation of the blank.

## 4. Conclusions

An LPFG sensor was theoretically designed and practically realized by combining three sensitivity enhancement methodologies:

- The mode transition of a cladding mode by the deposition of an overlay with an RI greater than the fiber core RI;
- The turn-around point of a cladding mode;
- The enhancement of the evanescent field by reducing the fiber cladding diameter through chemical etching.

The theoretical simulation was used for the determination of the LPFG sensor’s ideal parameters: fiber diameter after etching, thickness of the higher index overlay and grating pitch. The sensor was consequently realized, with very good agreement between the experimental and theoretical spectral characteristics. The etched LPFG sensor was finally tested as a biosensor by performing a standard IgG/Anti-IgG immunoassay in a closed thermostated microfluidic flow cell.

After fitting the experimental data with a logistic curve, the LOD was calculated with three different protocols based on three times the standard deviation of the blank, on the maximum value of the residuals and on the average of the residuals. The calculated LODs, even if not considering the best one due to some deviations of the data from the theoretical curve, demonstrate the effectiveness of the applied methodology for sensitivity enhancement.

The developed sensitive LPFG biosensor, integrated into the temperature-controlled flow cell, has been demonstrated to be applicable to the detection of real samples, such as human serum: this compact and robust system was tested with a model assay (IgG/anti-IgG immunoassay), but a transfer to other real applications for the detection of other biomolecules can be envisaged. The future vision of the proposed sensor is the further development of the sensing system for a multiplexed application for the detection of multiple target molecules by writing, for example, different gratings in the same fiber.

## Figures and Tables

**Table 1:** Fiber and grating parameters.

Parameters	Values Used for Simulation
Core RI	1.44985
Cladding RI	1.44400
Core diameter	7.3 µm
Fiber diameter	124.6 µm
Index modulation (Δn)	1.6 × 10^−4^
Grating period (*Λ*)	220 µm
No. of grating planes	140
Grating length	30.58 mm

**Table 2:** Parameters of the Logistic curve fitting.

	Value	Error
A_1_	−0.14	0.25
A_2_	296	2790
x_0_	6.81 × 10^10^	4.27 × 10^12^
p	0.166	0.038
Adjusted R-Square	0.996	

**Table 3:** Comparison among different LPFG-based biosensors for IgG/anti-IgG detection.

Kind of Sensor	Immobilized Receptor (Concentration)	Antigen	Measurement Setup	LOD	Ref.
LPFG	Anti-IgG (0.5 mg/mL)	IgG	Flow cell	Not given	[29]
Reflection mode LPFG	IgG (0.050 mg/mL)	Anti-IgG	Dip coating	Not given	[30]
LPFG in MT + overlay (polystyrene)	IgG (0.1 mg/mL)	Anti-IgG	Dip coating	Not given	[31]
LPFG at TAP	IgG (1 mg/mL)	Anti-IgG	Flow cell	7 × 10^−2^ μg/mL	[32]
Graphene oxide nanosheets functionalized dual-peak LPFG	IgG (1 mg/mL)	Anti-IgG	Dip coating	7 × 10^−3^ µg/mL	[33]
LPFG by laser ablation + overlay (tin dioxide)	IgG (2.4 mg/mL)	Anti-IgG	Dip coating	Not given Sensitivity: 1.1 nm/(mg/L)	[34]
Graphene-oxide-coated U-bent LPFG in a two-mode fiber	Anti-IgG	IgG	Dip coating	2 × 10^−2^ µg/mL	[35]
LPFG at the lowest-order CM and near TAP	IgG (1 mg/mL)	Anti-IgG	Flow cell	2 × 10^−4^ µg/mL	[36]
LPFG coated with PAH, SiO_2_ nanoparticles and gold nanoparticles	Anti-IgG (1 mg/mL)	IgG	Dip coating	Minimum detected concentration 10 µg/ml	[37]
LPFG coated with PAH, SiO_2_ nanoparticles and gold nanoparticles	Peptide	IgG	Flow cell	16.8 pg/mm^2^ (lowest conc. tested 1 × 10^−4^ µg/mL	[38]
LPFG near MT and near TAP + overlay (poly-cation and the poly-anion multiple layers)	IgG (1 mg/mL)	Anti-IgG	Flow cell	5 × 10^−4^ µg/mL 4.5 × 10^−6^ µg/mL	This work

## Data Availability

The data presented in this study are available on request from the corresponding author upon reasonable request.
